# Prevalence and temporal trend of prematurity in Brazil before and during the COVID-19 pandemic: a historical time series analysis, 2011-2021

**DOI:** 10.1590/S2237-96222023000200005

**Published:** 2023-05-19

**Authors:** Marcos Alberton, Vanessa Martins Rosa, Betine Pinto Moehlecke Iser

**Affiliations:** 1 Universidade do Sul de Santa Catarina, Curso de Medicina, Tubarão, SC, Brazil; 2 Universidade do Sul de Santa Catarina, Programa de Pós-Graduação em Ciências da Saúde, Palhoça, SC, Brazil

**Keywords:** COVID-19, Preterm Birth, Premature Baby, Time Series Studies, Brazil, COVID-19, Nacimiento Prematuro, Recien Nacido Prematuro, Estudios de Series Temporales, Brasil, Covid-19, Nascimento Prematuro, Recém-Nascido Prematuro, Estudos de Séries Temporais, Brasil

## Abstract

**Objective:**

to measure the prevalence of prematurity according to the Brazilian macro-regions and maternal characteristics over the past 11 years; to compare the proportions during the COVID-19 pandemic (2020-2021) with those of the historical series (2011-2019).

**Methods:**

this was an ecological study, with data from the Live Birth Information System; the prevalence was calculated according to year, macro-region and maternal characteristics; time series analysis was performed using Prais-Winsten regression model.

**Results:**

the prevalence of preterm birth in 2011-2021 was 11.1%, stable; the average in the pandemic period 11.3% (95%CI 11.2;11.4%) was similar to that of the base period 11.0% (95%CI 10.6;11.5%); the North region (11.6%) showed the highest proportion between 2011 and 2021; twin pregnancy (56.3%) and pregnant women who had 4-6 prenatal care visits (16.7%) showed an increasing trend (p-value < 0.001); the highest prevalence was observed for extremes of maternal age, pregnant women of Black race/skin color, indigenous women and those with lower level of education.

**Conclusion:**

preterm birth rates were highest for socially vulnerable pregnant women, twin pregnancies and in the North; stable prevalence, with no difference between periods.

## Resumo

**Objetivo:**

mensurar a prevalência de prematuridade segundo macrorregião brasileira e características maternas, nos últimos 11 anos; comparar as proporções durante a pandemia de covid-19 (2020-2021) com as da série histórica (2011-2019).

**Métodos:**

estudo ecológico, com dados do Sistema de Informações sobre Nascidos Vivos; prevalências calculadas segundo ano, macrorregião e características maternas; análise da série temporal pelo modelo de Prais-Winsten.

**Resultados:**

a prevalência de prematuridade em 2011-2021 foi de 11,1%, estável; a média no período pandêmico 11,3% (IC_95%_ 11,2;11,4%) assemelhou-se à do período-base 11,0% (IC_95%_ 10,6;11,5%); a região Norte (11,6%) apresentou a maior proporção entre 2011 e 2021; gestação gemelar (56,3%) e gestantes com 4-6 consultas de pré-natal (16,7%) apresentaram tendência crescente (p-valor < 0,001); observou-se maior prevalência para extremos de idade materna, gestantes de raça/cor da pele preta, indígenas e menor escolaridade.

**Conclusão:**

maior prematuridade nas gestantes socialmente vulneráveis, em gestações gemelares e no Norte; prevalência estável, sem diferença entre períodos.

## Resumen

**Objetivo:**

medir la prevalência de prematuridad de 2011 a 2021, según macrorregión brasileña y características maternas, comparando los años de pandemia de COVID-19, con 2011-2019.

**Métodos:**

estudio ecológico basado en el Sistema de Información de Nacimientos Vivos. Prevalencias calculadas según año, macrorregión y características maternas. Análisis de series temporales por el modelo generalizado de Prais-Winsten.

**Resultados:**

la prematuridad entre 2011-2021 fue 11,1%, con estabilidade; la media de en el periodo de la pandemia 11,3% (IC_95%_ 11,2;11,4) se asemejó a la media de referencia 11,0% (IC_95%_ 10,6;11,5). Región del norte tuvo la proporción más alta entre 2011 y 2021. Embarazo gemelar y mujeres con 4-6 visitas prenatales tuvieron tendencia al aumento. Prevalencias más elevadas correspondían a la edad materna extrema, mujeres embarazadas negras e indígenas y niveles de educación más bajos.

**Conclusión:**

mayor prematuridad en situación de vulnerabilidad social, embarazos gemelares y de la Región Norte. Proporciones se mantuvieron estables, sin diferencias entre períodos.

**Table:** 

Study contributions	
**Main results**	The prevalence of prematurity was 11.1% in Brazil in 2011-2021. It was stable, and no difference was found during the pandemic ; the North region showed the highest prevalence; increasing trend in the prevalence was observed for twin pregnancies and pregnant women who had 4-6 prenatal care visits.
**Implications for services**	Estimating the proportion of prematurity in Brazil and the influence of the COVID-19 pandemic on it contributes to improvement in health care aimed at the most vulnerable population and stimulates specific actions to prevent this indicator that burdens the health system.
**Perspectives**	There was no immediate effect of the COVID-19 pandemic on prematurity. Prenatal care for more vulnerable groups, such as pregnant women ≥ 40 years, those with lower level of education, indigenous women, twin pregnancies, and < 6 prenatal care visits, should be prioritized.

## INTRODUCTION

Preterm birth, defined as all “births before the 37^th^ week of pregnancy, or fewer than 259 days from the first date of a woman’s last menstrual period”, is a serious and growing health problem worldwide.^1^ ^),(^ ^2^ Recent data from the World Health Organization (WHO) show that globally, the prevalence of premature births ranges from 5% to 18%, and that every year an estimated 15 million babies are born preterm.^1^ This high occurrence of prematurity generates high socioeconomic costs and it is one of the leading causes of neonatal mortality.^1^ ^),(^ ^3^

Between 2011 and 2019, approximately 3 million preterm births were reported in Brazil, whose prevalence was 11%,^4^ ranking the country among the top ten countries with the highest occurrence of preterm births in the world.^1^ The Northeast and Southeast regions of Brazil concentrated 28% and 39% of these births, respectively, being the regions with the highest number of preterm births in the period from 2011 to 2019.^4^ The analysis of the maternal characteristics of these records revealed a higher frequency in pregnant women at extremes of age (≥ 40 years; < 15 years), with fewer than seven prenatal care visits and less than eight years of schooling.^5^

Social, environmental and maternal factors, such as air pollution, twin pregnancy, history of pregnancy complications, bacterial and/or viral infections, are associated with a higher risk of preterm birth.^6^ A multicenter study conducted in 20 Brazilian obstetric centers between April 2011 and July 2012 showed that among 1,084 pregnancies with indication for premature termination, the main complications influencing this outcome were preeclampsia (58.2%), chronic arterial hypertension (15.3%) and non-obstetric infections (1.5%).^7^ Among maternal infections, it is worth mentioning COVID-19, a disease caused by a new coronavirus (SARS-CoV-2), and its possible influence on preterm birth, either due to pathological issues of induction of labor before term, or pregnancy termination due to maternal complications of infection, such as respiratory syndrome.^8^ A meta-analysis that included 16 observational studies and 44 case reports, totaling 920 neonates of SARS-CoV-2- infected pregnant women, showed that approximately 37% of these deliveries were preterm births.^9^

By July 2022, Brazil had exceeded 30 million COVID-19 cases, second position in the ranking of countries with the highest number of infected people, and more than 600,000 deaths due to the disease.^10^ In this unfavorable epidemiological scenario for pregnant women, an analytical ecological study on the vulnerability of pregnant and puerperal women in Santa Catarina, conducted at the beginning of the pandemic, showed that areas where there is a higher proportion of teenage pregnancy, poor access to health services and low level of education are associated with municipalities that are less structured to tackle the emergence of COVID-19.^11^ These areas, common in a heterogeneous country such as Brazil, need support for serious conditions due to the infection, and may present frequent maternal-fetal complications.

Taking into consideration the impact of prematurity on newborn morbidity and mortality, with possible permanent sequelae, and the high costs to the health system, information on its occurrence is essential for organizing health care, especially for the most vulnerable groups. In the study by Martinelli et al.,^5^ the proportion of prematurity in Brazil showed a decreasing trend from 2012 to 2019, with variations according to maternal characteristics. In view of the emergence of the COVID-19 pandemic as of 2020, the inadequate control policy and possible influence of the virus on the number of premature births, this study aimed to measure the prevalence of prematurity in Brazil, according to macro-region and maternal characteristics, over the past 11 years, and compare the proportions during the SARS-CoV-2 pandemic, 2020 and 2021, with those of the historical series from 2011 to 2019, pre-pandemic period.

## METHODS

An ecological, time-series, before- and - after study was conducted using records from the Brazilian Live Birth Information System (*Sistema de Informações sobre Nascidos Vivos* - SINASC), from 2011 to 2021. Babies born preterm (< 37 weeks of gestation) were identified for the analysis of the longitudinal component of the time series, comprising the prevalence in the period from 2011 to 2021, and the prevalence between the periods of 2011-2019 (pre-pandemic) and 2020-2021 (pandemic) was compared, according to maternal characteristics and macro-regions.

Data from the 2010 Population Census show a total female population of reproductive age (age group 10-49 years) corresponding to 33% (62,110,637) of the Brazilian population.^12^ A total of 34,559,375 live births were registered in Brazil in the period from 2010 to 2021, of whom 5,402,191 (15.6%) in the pandemic period (2020 and 2021).^13^ The study population was comprised of all Brazilians born alive between 2011 and 2021.

The dependent variable of the study was prematurity, stratified according to the macro-region of residence (North; Northeast; Midwest; South; Southeast) and maternal and gestational characteristics, such as:

a) mother’s age (in full years: 10 to 19; 20 to 29; 30 to 39; 40 and older);
b) schooling (in complete years of study: 1 to 3; 4 to 7; 8 to 11; 12 or more);
c) mother’s race/skin color (White; Black; mixed race; Asian; indigenous);
d) type of pregnancy (single; twin);
e) number of prenatal care visits (1 to 3; 4 to 6; 7 or more); and
f) type of delivery (vaginal; cesarean section).

The independent variable was the time/year of registration of the collected information (2011 to 2021). Quality prenatal care was considered based on the current guidelines defined by the Ministry of Health, that is, having at least six prenatal care visits.^14^

Data for the period 2011 to 2020 were extracted from SINASC, made available via TABNET, a DATASUS tool, while those for 2021 were retrieved from the Live Birth Monitoring Panel, given the unavailability of SINASC data update in TABNET. Both procedures used the CSV file extension to MS-Excel and were tabulated according to the variables of interest.^4^ ^),(^ ^13^

The proportions of prematurity were calculated by dividing the number of preterm births (< 37 weeks of gestation), in each category of the variables of interest and year, by the total number of live births for the same category and period, in the same place and period, multiplied by 100.

In order to evaluate the time series, we used Prais-Winsten regression model, taking into consideration robust standard errors to model heteroscedasticity according to the structure of variance-covariance matrix. To verify the presence of serial autocorrelation, the Durbin-Watson hypothesis test was applied, and values close to 2 were expected as indicative of absence of serial autocorrelation. The value of the angular coefficient (β), positive or negative, represents, respectively, the average annual increase or decrease in the proportions of prematurity for each year analyzed. Based on that, it was also verified whether the trend was stationary, by means of the hypothesis test for the estimated coefficient, i.e.: a stable trend is assumed in H0 and, if H0 is not rejected (p-value ≥ 0.05), the trend is considered as stationary; when H0 is rejected (p-value < 0.05), the trend is considered as descending (negative regression coefficient) or ascending (positive regression coefficient), in each category of the variables studied. For statistical comparison of proportions among categories, the period average and 95% confidence intervals (95%CI) were taken into consideration, and they should not overlap. The significance level was 5%. The average prevalence of the pandemic period (2020-2021) was compared to the average prevalence of the previous period (2011-2019), according to the macro-regions of Brazil, maternal and gestational characteristics, with respective 95%CI. The percentage change was calculated as follows:

[(pandemic prevalence - pre-pandemic prevalence)*100]/(pre-pandemic prevalence)

The results were analyzed using the Stata 12.0.^15^

As this is an ecological study without individual data and using publicly available data, the study project does not meet the conditions required for registration and analysis by a Research Ethics Committee.^16^

## RESULTS

In Brazil, a total of 31,625,722 live births were reported between 2011 and 2021. Of these, 3,503,085 (11.0%) were premature, and the prevalence of prematurity was 11.1%. The North region showed the highest proportion of prematurity, 11.6% (95%CI 11.2; 12.1), higher than that of the Midwest region, 10.8% (95%CI 10.6;11.1), which was the lowest proportion in the period (Table 1). When evaluating the trend in prematurity in Brazil and by macro-region throughout the study period, from 2011 to 2021, stability was evidenced, despite small numerical variations year by year.

**Table 1:** Prevalence of prematurity and time trend, Brazil and macro-regions, 2011-2021

Brazil and macro-regions	Average prevalence (%)	Point prevalence (%)			Trend from 2011 to 2021			
	2011-2021	95%CI^a^	2011	2021	β Coeff^b^	95%CI^a^	p-value	Interpretation
Brazil	11.1	10.7;11.4	9.8	11.3	0.46	-0.85;1.77	0.448	Stable
North	11.6	11.2;12.1	9.9	11.8	0.78	0.96;2.53	0.338	Stable
Northeast	11.0	10.8;11.2	10.5	11.2	0.16	-0.63;1.05	0.656	Stable
Southeast	10.9	10.5;11.3	9.3	11.3	0.72	-0.48;1.93	0.209	Stable
South	11.1	10.6;11.5	9.4	11.3	0.47	-1.34;2.28	0.570	Stable
Midwest	10.8	10.6;11.1	10.0	11.3	0.69	-0.22;1.58	0.114	Stable

a) 95%CI: 95% confidence interval; b) β Coeff: Beta coefficient of the regression, indicating the variation in percentage points per year.

Regarding maternal characteristics, it could be seen that the average prevalence of prematurity (Table 2) varied with maternal age, in the period from 2011 to 2021. Extremes of age groups (10 to 19 years old; 30 years and older) showed higher risk of preterm birth; pregnant women aged 40 years and older had the highest prevalence, 14.9% (95%CI 14.5;15.4); pregnant women aged 20 to 29 years showed lower proportion of prematurity 10.0% (95%CI 9.7;10.3), when compared to the other age groups. A higher prevalence of prematurity was evidenced among pregnant women with less than eight years of schooling, when compared to those with eight years of schooling or more.

**Table 2:** Prevalence of prematurity and time trend analysis according to maternal and gestational characteristics, Brazil, 2011-2021

Maternal and gestational characteristics	Average prevalence (%)	Point prevalence (%)			Trend from 2011 to 2021			
	2011-2021	95%CI^a^	2011	2021	β Coeff^b^	95%CI^a^	p-value	Interpretation
Mother’s age (in full years)								
10-19	12.6	12.2;13.0	11.2	12.4	-0.04	-1.76;1.67	0.954	Stable
20-29	10.0	9.7;10.3	8.9	10.1	0.23	-1.01;1.48	0.677	Stable
30-39	11.6	11.2;12.0	10.3	12.2	0.86	-0.35;2.07	0.141	Stable
≥ 40	14.9	14.5;15.4	13.2	15.8	1.56	0.13;3.00	0.095	Stable
Mother’s schooling (in complete years of study)								
1-3	12.9	12.4;13.4	10.9	13.8	1.47	-0.11;3.05	0.065	Stable
4-7	12.2	11.8;12.6	10.4	12.7	0.99	-0.67;2.66	0.211	Stable
8-11	10.7	10.4;11.1	9.4	11.1	0.64	-0.61;1.89	0.277	Stable
≥ 12	10.8	10.5;11.2	9.6	11.1	0.48	-0.56;1.53	0.324	Stable
Mother’s race/skin color								
White	11.0	10.6;11.4	9.3	11.3	0.71	-0.81;2.23	0.316	Stable
Black	11.8	11.6;12.1	11.7	11.7	-0.62	-1.43;0.18	0.110	Stable
Mixed race	11.0	10.7;11.3	10.0	11.1	0.27	-0.97;1.50	0.631	Stable
Asian	11.2	10.9;11.6	10.0	11.6	0.71	-0.75;2.37	0.300	Stable
Indigenous	14.4	13.7; 15.0	14.3	14.2	-0.59	-4.07;2.88	0.706	Stable
Type of pregnancy								
Single	10.1	9.8;10.4	9.0	10.2	0.17	-1.16;1.51	0.078	Stable
Twin	56.3	54.1;58.6	50.5	61.2	9.81	8.82;10.81	< 0.001	Increase
Number of prenatal care visits								
1-3	21.8	21.3;22.3	196.3	21.9	1.15	-1.02;3.33	0.260	Stable
4-6	16.7	15.9;17.6	136.2	18.5	3.54	2.13;4.97	< 0.001	Increase
≥ 7	8.0	7.7;8.4	6.7	8.5	0.82	-0.36-2.01	0.149	Stable
Type of delivery								
Vaginal	11.0	10.6;11.4	10.0	10.6	-0.52	-2.20;1.15	0.498	Stable
Cesarean section	11.1	10.7;11.5	9.7	11.9	1.23	0.13;2.34	0.093	Stable

a) 95%CI: 95% confidence interval; b) β Coeff: Beta coefficient of the regression, indicating the variation in percentage points per year.

The prevalence was higher in pregnant women of indigenous race/skin color (14.4%), when compared to other ethnic groups. Black pregnant women had a significantly higher prevalence when compared to the white and mixed-race categories. Pregnant women who had more than seven prenatal care visits showed the lowest proportion of prematurity in the study 8.0% (95%CI 7.7;8.4), compared to those with fewer prenatal care visits; those who had between four and six prenatal care visits showed an increasing trend in prematurity over the study period. No association was found between prematurity and vaginal delivery or cesarean delivery, maintaining stability in the trend of prematurity in both types of delivery, in the period from 2011 to 2021.

The prevalence of prematurity in single pregnancies was 10.1% (95%CI 9.8;10.4), while the prevalence in twin pregnancies was 56.3% (95%CI 54.1;58.6) in the period from 2011 to 2021. The variable “twin pregnancy”, therefore, showed the greatest association with prematurity, when compared to any other maternal characteristic. In addition, the average prematurity in twin pregnancies showed an increasing trend of 9.81 percentage points/year (95%CI 8.82;10.81) in the period from 2011 to 2021.

Taking into consideration the pre-pandemic period as the baseline, the average prevalence of prematurity was 11.0% in Brazil (95%CI 10.6;11.5), while in the pandemic period, the average prevalence of prematurity was 11.3% (95%CI 11.2;11.4). The North region had the highest proportion for both periods, although in the pandemic period, the prevalence was 11.9% (95%CI 11.5;12.2) in this region, higher than the national average. The Midwest showed the lowest proportion among the macro-regions in the country in the pre-pandemic period, 10.8% (95%CI 10.5;11.0); in the pandemic period, the Northeast region showed the lowest prevalence, 11.2% (95%CI 10.6;11.8) (Table 3).

**Table 3:** Prevalence of prematurity according to national macro-region, mother and gestational characteristics, comparing the pre-pandemic and pandemic periods, Brazil, 2011-2021

Variables	Average prevalence (%) Pre-pandemic period: 2011-2019	95%CI^a^	Average prevalence (%) Pandemic period: 2020-2021	95%CI^a^
Brazil	11.0	10.6;11.5	11.3	11.2;11.4
North	11.6	11.0;12.1	11.9	11.5;12.2
Northeast	11.0	10.8;11.2	11.2	10.6;11.8
Southeast	10.8	10.3;11.3	11.3	11.0;11.6
South	11.0	10.4;11.6	11.3	10.7;11.9
Midwest	10.8	10.5;11.0	11.2	10.9;11.6
Mother’s age (in full years)				
10-19	12.6	12.1;13.1	12.5	11.6;13.3
20-29	10.0	9.5;10.4	10.1	10.0;10.2
30-39	11.5	11.1;11.9	12.1	11.5;12.8
≥ 40	14.8	14.3;15.3	15.7	14.6;16.8
Mother’s schooling (in complete years of study)				
1-3	12.7	12.2;13.3	13.6	10.9;16.3
4-7	12.1	11.6;12.6	12.6	12.1;13.1
8-11	10.7	10.2;11.1	11.1	11.0;11.2
≥ 12	10.8	10.4;11.2	11.1	10.9;11.3
Mother’s race/skin color				
White	10.9	10.4;11.4	11.3	10.8;11.8
Black	11.8	11.5;12.2	11.7	11.6;11.8
Mixed race	11.0	10.6;11.3	11.2	10.9;11.4
Asian	11.1	10.7;11.6	11.7	10.9;12.5
Indigenous	14.4	13.6;15.3	14.1	12.7;15.4
Type of pregnancy				
Single	10.1	9.6;10.5	10.2	10.1;10.3
Twin	55.4	53.2;57.5	60.8	54.9;66.7
Number of prenatal care visits				
1-3	21.9	21.2;22.5	21.6	17.9;25.3
4-6	16.4	15.5;17.4	18.0	12.7;23.4
≥ 7	7.9	7.5;8.3	8.5	8.0;8.9
Type of delivery				
Vaginal	11.1	10.5;11.6	10.7	9.4;12.0
Cesarean section	11.0	10.6;11.4	11.8	10.7;12.9

a) 95%CI: 95% confidence interval.

The comparison of the average prevalence of prematurity observed between the periods showed no statistically significant differences according to maternal and gestational characteristics. However, the greatest variations in the last period occurred in pregnant women aged 40 years and older (6.1%), in those with 1 to 3 years of schooling (7.1%), of Asian race/ skin color (5.4%), in twin pregnancies (9.7%), among women who had 4 to 6 prenatal care visits (9.8%), and in cesarean section (7.3%) 3 and 4 (Table 4).

**Table 4:** Annual prevalence (%) of prematurity according to national macro-region, mother and gestational characteristics, Brazil, 2011-2021

Variables	2011	2012	2013	2014	2015	2016	2017	2018	2019	2020	2021
Brazil	9.8	11.9	11.5	11.2	10.8	11.1	10.9	11.0	11.1	11.3	11.3
North	9.9	12.5	12.1	11.8	11.4	11.5	11.4	11.6	12.1	11.9	11.8
Northeast	10.5	11.3	11.3	11.1	10.9	11.3	10.9	10.8	10.8	11.3	11.2
Southeast	9.4	12.2	11.7	11.2	10.8	11.0	10.9	11.0	11.0	11.2	11.3
South	9.3	11.8	11.0	10.9	10.6	11.0	10.8	11.0	11.1	11.2	11.3
Midwest	10.0	11.3	11.0	10.8	10.7	10.7	10.5	10.9	11.0	11.2	11.3
Mother’s age (in full years)											
10-19	11.2	13.6	13.3	12.9	12.5	12.8	12.5	12.4	12.4	12.5	12.4
20-29	8.9	10.8	10.4	10.1	9.8	10.1	9.8	9.9	9.9	10.1	10.1
30-39	10.3	12.3	11.8	11.5	11.3	11.6	11.5	11.6	11.7	12.1	12.2
≥ 40	13.2	15.4	15.1	14.7	14.5	14.7	14.9	15.1	15.4	15.6	15.8
Mother’s schooling (in complete years of study)											
1-3	10.9	13.3	13.0	12.9	12.6	13.1	12.9	13.1	12.7	13.4	13.8
4-7	10.4	12.9	12.6	12.3	12.0	12.3	12.1	12.2	12.2	12.6	12.7
8-11	9.4	11.4	11.1	10.8	10.4	10.8	10.7	10.7	10.8	11.1	11.1
≥ 12	9.6	11.6	11.0	10.9	10.7	10.9	10.6	10.8	11.0	11.1	11.1
Mother’s race/skin color											
White	9.3	11.9	11.4	11.0	10.7	11.0	10.9	11.0	11.1	11.3	11.3
Black	11.7	12.7	12.3	12.0	11.5	11.9	11.5	11.5	11.5	11.7	11.7
Mixed race	10.0	11.8	11.5	11.1	10.8	11.0	10.8	10.9	11.0	11.2	11.1
Asian	10.0	11.8	11.7	11.4	11.0	11.4	10.5	11.2	11.4	11.7	11.6
Indigenous	14.3	15.9	16.1	15.2	14.4	14.0	13.5	12.9	13.6	14.0	14.2
Type of delivery											
Single	9.0	11.0	10.6	10.3	9.9	10.2	9.9	9.9	10.0	10.2	10.2
Twin	50.5	53.8	53.2	54.6	54.7	56.3	57.1	58.6	59.5	60.3	61.2
Number of prenatal care visits											
1-3	19.6	22.4	21.9	21.8	21.7	22.2	22.3	22.3	22.3	21.3	21.9
4-6	13.6	16.4	16.1	16.1	16.2	16.9	17.1	17.5	18.1	17.6	18.5
≥ 7	6.7	8.6	8.2	8.0	7.7	8.0	7.8	8.0	8.2	8.5	8.5
Type											
Vaginal	9.9	12.2	11.8	11.5	10.9	11.1	10.8	10.7	10.6	10.8	10.6
Cesarean section	9.7	11.6	11.2	11.0	10.8	11.2	11.1	11.2	11.4	11.7	11.9

## DISCUSSION

In Brazil, the prevalence of prematurity was 11.1% in the period from 2011 to 2021, and showed a stable trend. In the North region, pregnant women aged 40 years and older, those with lower level of education, being an indigenous woman, women who had fewer than eight prenatal care visits and those with twin pregnancies showed the highest prevalence. Even pregnant women who had 4 to 6 prenatal care visits and women with twin pregnancies showed an increasing trend in the period, the latter - twin pregnancy - being the variable that showed the greatest relationship with prematurity.

Stability in prematurity was observed for all macro-regions. However, the North region showed a significantly higher prevalence of prematurity than the Midwest in the period from 2011 to 2021, with the two macro-regions having the highest and the lowest prevalence, respectively. The North region also showed prevalence of prematurity significantly higher than the national average in the 2020-2021 biennium. There was no statistically significant difference when comparing the pandemic and pre-pandemic periods, both when analyzing maternal characteristics and by macro-region.

A systematic review published in The Lancet journal in 2019, when evaluating the global prematurity rates in 2014, showed that Brazil ranked ninth among the countries with the highest number of preterm births, at a proportion of 11.2% - a value similar to that found in this study. The frequency that the study attributed to Brazil, although higher than the world standard (10.6%) in 2014, showed stability compared to 2000, while for the world, according to the same study, the prevalence of prematurity increased from 9.8% (95%CI 8.3;10.9) in 2000 to 10.6% (95%CI 9.0;12.0) in 2014.^17^

An analysis of the Midwest region showed an increasing trend in the period from 2000 to 2019, with a 0.5% annual growth (95%CI 0.37;0.75),^18^ unlike the stability showed in this study with the inclusion of the years 2020 and 2021 in the analysis. The significantly higher prevalence in the North region compared to the national average, in the pandemic period, has already been mentioned in a national publication, which pointed out, as possible justifications, important risk factors such as teenage pregnancy, low level of education and inadequate prenatal care in the region.^19^

Based on the results of this study, it is possible to suggest that prematurity is related to inadequate prenatal care, since higher prevalence was found in pregnant women who had one to three prenatal care visits, followed by those who had four to six visits. These data are in line with the national literature, according to which females who undergo prenatal care are less likely to have preterm births and that the lower the number of prenatal care visits, the higher the proportions of prematurity.^20^ In the present evaluation, however, it is worth highlighting that the anticipation of childbirth leads to a reduction in the number of prenatal care visits, especially because the frequency of prenatal care increases at the end of the gestation period. A national study showed an increase in prenatal care adequacy rates in the period from 2012 to 2018, although there is still stability in prematurity.^19^

The increase in prematurity in pregnancies with four to six prenatal care visits may be related to impairment of quality of visits, or even to the impact of the COVID-19 pandemic, which limited health care aimed at reducing the spread of the virus, especially for risk groups, such as pregnant women.^21^ It can be seen that, although most pregnant women receive prenatal care, a large proportion of them do not undergo all the procedures recommended by the Ministry of Health. Thus, the identification of gestational risk by health professionals and appropriate referral are impaired, and may increase the occurrence of maternal and perinatal morbidity and mortality outcomes.^5^ It should be taken into consideration that, when a high-risk pregnancy is identified, it is possible to recommend more prenatal care visits for the most appropriate monitoring of pregnancy.^18^

As found worldwide^6^ and in Brazilian studies with small populations, a discrepancy in prematurity was found when comparing different races and ethnicities.^22^ In the United States, in 2015, non-Hispanic black pregnant women had a two-fold greater risk for preterm birth, compared with non-Hispanic white women.^23^

In this study, prematurity in the Brazilian indigenous population was higher than those found among the white, black, Asian and mixed-race populations, with no difference among the last four populations. This finding corroborates the results of a Brazilian cohort study conducted with the Guarani indigenous population, whose prevalence of prematurity was higher than the national average.^24^ These differences may be related to the quality of prenatal care offered to the indigenous population, or even to the access to health services among this population. Over the last two decades, the Indigenous Health Care Subsystem of the Brazilian National Health System (*Subsistema de Atenção à Saúde Indígena do Sistema Único de Saúde* - SASISUS) has been implemented, aiming at improving prenatal care and expanding the coverage of prenatal care visits in the villages; however, the quality of prenatal care for indigenous women remains below that provided to the general Brazilian population, in relation to the start date of prenatal care, number of prenatal care visits, clinical and laboratory follow-up.^24^

Prematurity tends to decrease with an increase in maternal education,^6^ ^),(^ ^20^ being considered an independent causal factor for preterm birth.^25^ In this study, this association is evident, given that pregnant women with less than eight years of schooling had a higher frequency of preterm babies, when compared to those with eight years of schooling or more.

Pregnant women aged between 20 and 29 years had a lower number of preterm births. On the other hand, people at the extremes of age (under 20 and 30 years and older) showed a higher prevalence of prematurity, corroborating the results of the international and national literature.^6^ ^),(^ ^20^ ^),(^ ^25^ National studies conducted between 2011 and 2012 showed an increase in this occurrence in pregnant women aged 35 years and older and those under 19 years of age.^7^ ^),(^ ^20^ After all, this population has a higher frequency of gestational comorbidities, such as diabetes *mellitus*, arterial hypertension, preeclampsia, which are considered risk factors for prematurity.

Equally important is to highlight the change in fertility among Brazilian pregnant women, who are delaying their first pregnancy. Thus, prematurity is expected to increase over the next few years,^26^ even if there was no significant increase in prematurity for pregnant women aged 40 years and older, from 2011 to 2021.

Furthermore, women aged 30 years and older have a higher number of infertility treatments and therefore have more multiple pregnancies, which, as observed in this study, impact on the proportion of prematurity.^7^ The increase in prematurity in multiple pregnancies is unanimous evidence in the literature and, according to the results of the present study, this increased by two to 40 times, which is also related to the number of fetuses.^7^ ^),(^ ^20^ This is due to the fact that multiple pregnancy is a condition of higher risk for the fetuses, and the intrauterine period influences this risk. It is worth mentioning the trend of increased prematurity associated with the variable “twin pregnancy”, over the entire period (2011-2021), possibly related to the increase in infertility treatments in recent years and its direct impact on twin pregnancies.^7^

The type of delivery - natural birth or cesarean section - was not associated with prematurity, unlike other studies in which cesarean section was a variable associated with deliveries prior to 37 weeks of gestation. Although this surgery increases maternal and perinatal morbidity and mortality, the risks inherent to preterm labor, or other conditions that cause spontaneous pregnancy termination or recommended by health professionals, justify the increase in cesarean section as a necessary therapy in some cases.^7^ In this study, there was stability of prematurity in both routes of delivery, from 2011 to 2021.

In addition to these findings, it is worth mentioning the relevance of the comparison between the periods 2011-2019 and 2020-2021, taking into account that the WHO declared the SARS-Cov-2 a pandemic on March 11, 2020 and the implication of this infection on pregnant women and fetuses.^27^ When comparing the proportions of prematurity in Brazil, between the pre-pandemic (2011-2019) and pandemic (2020-2021) periods, the small increase observed in the prevalence of the general population, from 11.0% (95%CI 10.6;11.5) to 11.3% (95%CI 11.2;11.4), showed no statistically significant difference. This finding is consistent with that found by Wilkinson et al. in a cohort study conducted in England between January 2016 and January 2021, when no significant variation in prematurity was observed, although symptomatic pregnant women were related to preterm births due to iatrogenic, maternal indications and practice of emergency obstetric.^28^

A Danish retrospective register-based study, aimed at evaluating the effectiveness of lockdown - in the period from March 14, 2020 to September 30, 2020 - showed a protective effect on extremely preterm infants, with a 70% reduction in the prevalence; however, for premature infants in general, a 20% reduction was observed, with no evidence of this protective factor.^29^

The increase in preterm births in the pandemic period was a result expected by these authors, given that the association between COVID-19, as well as its family of viruses,^30^ and preterm births was found in the literature in up to 37% of births. This result is probably related to the pregnancy termination due to the severity of pneumonia in pregnant women.^9^ In this study, however, the comparison between the periods showed no significant difference, perhaps due to the short time of evaluation of the pandemic period (2020-2021), and the influence of interruption of prenatal care may appear late; or, due to the ecological approach of the study that did not evaluate specific cases of maternal infection during pregnancy.

Other limitations of this study are related to the use of secondary data, dependent on the quality and completeness of SINASC data (probable losses regarding the characteristics “race/skin color” and “schooling”), in which existing data and categories standardized by the Ministry of Health were analyzed. Furthermore, this is a population-based study, therefore the relationships observed among the population characteristics may neither represent associations nor causality at the individual level. In order to avoid ecological fallacy biases, data on the outcome “prematurity” were interpreted according to the pathophysiology and previously known and related causes. It is noteworthy that an increase in the number of prematurity cases between 2011 and 2012 may have occurred due to changes in the content of the Certificate of Live Birth (COLB) and a decrease in underreporting.

This study showed that the highest prevalence of prematurity during the entire period, from 2011 to 2021, corresponded to pregnant women aged 40 years and older, lower level of education, indigenous women, having fewer than eight prenatal care visits and twin pregnancies, and a trend of increased prematurity for the period 2011-2021 was found in these last two categories. Probably, this group of pregnant women has greater difficulty accessing health care and inadequate filling in of prenatal care booklets. The North region had the highest prevalence of prematurity in the country in the entire period. Twin pregnancies or pregnancies that had up to six prenatal care visits showed a trend of increasing prematurity for the period from 2011 to 2021. Finally, overall prematurity remained stable during the study period, with no difference when comparing the averages in the pre-pandemic and pandemic periods.

The results of this study reinforce the need to improve health care for pregnant women, especially those belonging to segments of greater ethnic and social vulnerability, so that the mother-fetus binomial has the necessary high complexity health care for this condition. By highlighting the variables associated with prematurity, the development of assertive prevention policies is encouraged. Given that prematurity implies high costs for the health system, higher quality prenatal care, with emphasis on the most vulnerable groups and those with difficulties accessing health care, should be prioritized.
